# Rubric for peer evaluation of oral presentations: Use and perceptions among experienced and non‐experienced students

**DOI:** 10.1002/jdd.13831

**Published:** 2025-01-17

**Authors:** Juan José Pérez‐Higueras, Juan José Hidalgo Arroquia, Lucía Gancedo‐Caravia

**Affiliations:** ^1^ Department of Conservative and Prosthetic Dentistry Complutense University Madrid Spain

**Keywords:** communicative skills, peer assessment, rubrics, student learning

## Abstract

**Purpose:**

A properly designed rubric for oral presentations should be useful both to assess students’ performance and to help them prepare for the task. However, its use and perceptions might be influenced by scholars’ previous familiarization with rubrics during pre‐university courses. The aim of this study was to evaluate how the previous experience of students in the use of rubrics can influence their assessment of oral presentations and to compare their ratings with those assigned by educators.

**Methods:**

Eighty‐six first‐year undergraduate dentistry students were randomly distributed in teams to prepare oral presentations. A newly designed assessment rubric was presented to the students prior to the assignment. Six weeks later the students performed the presentations and were anonymously assessed with the rubric by their peers and seven educators (EDU). Students’ perceptions towards the rubric as a learning and assessment tool were registered with an anonymous survey, which also recorded if they were familiar with the use of rubrics (experienced students, ES) or not (not‐experienced students, NES). Assigned scores by NES, ES, and EDU were compared.

**Results:**

Sixty‐seven students completed the survey. No differences were found in the scores assigned among experienced (41) and non‐experienced students (26). Educators assigned significantly lower scores than students. ES and NES considered the rubric a complete easy to use and useful tool that helped them feel confident during assessment and performance.

**Conclusions:**

Previous experience does not influence students’ use and perceptions of the newly developed rubric, however, ratings assigned by students are not comparable to those of EDU.

## 1 INTRODUCTION

Acquisition of communicative skills is essential among health science students^1^, ^2^ as in their future professional practice they are expected to communicate effectively both with patients and with other professionals and researchers. Consequently, dentistry graduate and postgraduate programs should include this specific competence as part of their educational goals.^3^, ^4^

Providing specific instruction in the acquisition of communicative skills is a rather challenging task. It requires the incorporation of specific teaching‐learning activities for communication training among the learning methods of the different subjects in dentistry degrees. However, the educational practices in communication are not sufficiently standardized.^5^ Oral presentations performed by students in front of their peers have been considered pertinent activities, though often not effectively implemented.^6^

Ideally, procuring communication capability should be addressed from the constructive alignment (CA) approach. The CA philosophy introduced by Biggs proposes an overall correspondence in all the steps of the teaching‐learning process.^7^ This entails a logical connection between the statement of intended learning outcomes and the teaching‐learning activities, which should also be aligned with the assessment process. For an intended outcome of effective communication, learning activities such as oral presentations should be unmistakably aligned with a clear definition of the expected results of communicative proficiency and the specific criteria to assess the level of achievement accomplished by the students.

Appropriate assessment of these activities must fulfill two basic requirements: First, it should contemplate all the important aspects of communication to ensure an adequately aligned teaching‐learning process. At the same time, assessment should not be only summative, but also formative. Formative assessment provides feedback to the student about their level of achievement in a specific task and indicates how their performance could be improved to acquire the standard level required.^8^ Formative assessment is considered a useful strategy to help students learn more effectively and educators to accomplish the summative assessment in a more informed way.^8^

Novel focuses on assessment have proposed students as active players in the procedure. Assessment among peers has been put forward as a positive and effective evaluation practice^9^, ^10^, ^11^ and it has been shown to improve professionalism, interpersonal skills, and work habits among students.^12^

Rubrics are among the assessment tools that have been proposed to evaluate oral presentations.^13^, ^14^ They are documents that relate a list of ordered evaluation criteria previously established for a specific task with different explained levels of performance.^15^ These tools allow evaluators to determine the quality of a student's work based on precisely specified standards. With them, the student can compare their work with the required performance and comprehend the grounds for the score received. For this reason, rubrics have been widely incorporated in the formative assessment process.^16^ Previous studies have described several benefits of rubrics, such as enabling students to focus their efforts efficiently,^17^ helping them identify critical issues, and also allowing them to easily evaluate their own and their peers’ performance.^18^, ^19^, ^20^ The use of rubrics has also been shown to improve the perception of their assessment, as they tend to find their assigned scores better and fairer.^17^ Nevertheless, to optimize its benefits, some authors suggest that a previous familiarization of evaluators is needed to make the assessment process easier and smoother.^21^ For all these reasons, rubrics have been also implemented in pre‐university years.^22^

The combination of the active approach of peer assessment with the use of rubrics could be a suitable teaching‐learning activity to improve the learning results as well as the engagement of the students, taking advantage of the benefits of both strategies. However, not all students begin their university studies with a previous experience of being exposed to rubrics and assessed with them, a fact that might interfere with the proper use of these tools and with their impact on the students’ learning process. Thus, before implementing this assessment methodology, a relevant issue to address is to detect any influence that previous experience with rubrics could have on the student's use and perceptions of the tool, both as an assessment and as a learning instrument.

The aim of this study is to compare the influence of the previous experience during preuniversity courses of first‐year dentistry students in the use and perceptions of a newly designed rubric when preparing and assessing oral presentations. The null hypothesis tested was that there is no significant difference between experienced and non‐experienced students in the scores assigned in a peer assessment exercise and in students’ perceptions of the rubric as a learning tool or as an assessment tool. The study also aimed to compare the scores assigned by experienced and non‐experienced students with those assigned by educators. The second null hypothesis was that there is no significant difference between the scores assigned by either experienced or non‐experienced students in a peer assessment exercise and those assigned by educators using the same assessment rubric.

## 2 MATERIALS AND METHODS

This study was conducted in the Faculty of Dentistry of Complutense University with the approval of the Ethics Committee of Clinical Research of Clinical San Carlos Hospital (Madrid, Spain) (C.I. 23/733‐E).

### 2.1 Participants

The study was conducted within the cohort of first‐year undergraduate dental students enrolled in the subject Introduction to Dentistry, which is taught during the second half of the school year. Faculty members (educators [EDU]) with at least 5 years of experience in teaching and familiarized with the use of rubrics during assessment procedures also took part in it. A sample size calculation was performed to detect a difference in the scores (δ*) of 1, on a scale from 0 to 10, assuming a standard deviation (σ^2^) of 1.056, based on data obtained from a previous pilot study, using the following equation: n = (z_α/2_ + z_β_)^2^ * (2 σ^2^/ δ*^2^). An alpha (α) risk of 0.05 and a beta (β) risk of 0.10 in a bilateral contrast test were accepted. A minimum of twenty‐three participants per group were required to detect a difference (δ*) of 1 or higher. All 86 first‐year undergraduate students enrolled in the subject were invited to voluntarily participate after being informed of the aims and design of the study. The volunteers signed a written consent before their recruitment.

### 2.2 Preparation of the presentations

Students were randomly distributed into sixteen groups of 5–6 students. A different dentistry‐related topic, such as “Selection of dental biomaterials”, “Management of dental emergencies” or “Informed consent in dentistry” was assigned to each group and their members were requested to prepare an oral presentation that should last 10 min. A newly designed assessment rubric (Table 1) was presented before the preparation period in an interactive session in which the tool was explained to the students. The rubric consisted of nine items: four related to the content of the presentation (adequacy of the topic, organization, vocabulary, and documentation) and five related to the presentation display (support materials, presenter‐support materials interaction, verbal communication, non‐verbal communication, and presenter‐audience interaction). All items were to be graded according to four levels of performance (excellent, satisfactory, needs improvement, and needs much improvement).

**TABLE 1:** Newly designed assessment rubric for oral presentations in health science.

	Levels of performance			
	Excellent (4)	Satisfactory (3)	Needs improvement (2)	Needs much improvement (1)
**CONTENT**	**DOMAIN 1: ADEQUACY AND MASTERY OF THE TOPIC**			
	‐ RELEVANT content THOROUGHLY addressed. ‐ The presenter SHOWS MASTERY on the topic and is ABLE to EXPAND ON IT. ‐ The presenter answers ALL the questions appropriately and provides examples.	‐ The Content is RELEVANT. SOME DETAIL(s) is/are LEFT UNADDRESSED. ‐ The presenter KNOWS THE TOPIC and is ABLE to EXPAND ON PARTS OF IT but NOT on ALL POINTS. ‐ The presenter answers MOST of the questions appropriately and provides examples.	‐ VAGUE or INACCURATE content. ‐ The presenter SHOWS SOME KNOWLEDGE of the topic but IS UNABLE TO EXPAND ON IT. ‐ The presenter answers the questions VAGUELY or REPEATS the same information.	‐ INCORRECT content. ‐ The presenter DOES NOT KNOW the topic. ‐ The presenter is UNABLE to answer questions.
	**DOMAIN 2: ORGANIZATION**			
	‐ WELL‐ORGANIZED STRUCTURE and LOGICAL ORDER. ‐ The presentation is EASILY FOLLOWED	‐ ORGANIZED STRUCTURE with SOME UNCONNECTED SECTIONS. ‐ The presentation is easily FOLLOWED MOST OF THE TIME.	‐ LACKING AN ORGANIZED STRUCTURE and a LOGICAL ORDER ‐ It is DIFFICULT TO FOLLOW the presentation	‐ THERE IS NO STRUCTURE or LOGICAL ORDER. ‐ It is IMPOSSIBLE TO FOLLOW the presentation.
	**DOMAIN 3: VOCABULARY**			
	‐ ALWAYS uses technical language CORRECTLY. ‐ ADAPTS the vocabulary to the audience.	‐ Uses technical language CORRECTLY MOST OF THE TIME. ‐ OCCASIONALLY uses vocabulary that is CONFUSING for the audience.	‐ SIMPLE/INAPPROPRIATE language. ‐ The presenter DOES NOT DEFINE THE SPECIFIC TERMINOLOGY on the topic.	‐ INCORRECT/INAPPROPRIATE language. ‐ CONTINUOUSLY uses vocabulary that THE AUDIENCE DOES NOT UNDERSTAND.
	**DOMAIN 4: SOURCES OF INFORMATION (DOCUMENTATION)**			
	‐ The presenter uses VERIFIED DATA. ‐ ALL SOURCES used are RIGOROUS and WELL PRESENTED.	‐ MOST of the DATA are VERIFIED. ‐ MOST SOURCES are RIGOROUS and WELL PRESENTED.	‐ ONLY SOME DATA are VERIFIED by the bibliography. ‐ SOME of the SOURCES used are RIGOROUS and WELL PRESENTED.	‐ UNVERIFIED DATA. ‐ The SOURCES used are NOT RIGOROUS or ARE NOT IDENTIFIED.
**PRESENTATION**	**DOMAIN 5: SUPPORTING MATERIAL (TEXT, ICONOGRAPHY, ANIMATIONS, ETC.)**			
	‐ The SIZE and AMOUNT of TEXT are LEGIBLE and HELP to FOLLOW the presentation. ‐ ALL IMAGES are WELL‐CHOSEN, and their QUALITY and SIZE MAKE THEM VISIBLE. ‐ The order of appearance of ALL the elements CLARIFIES and HELPS to FOLLOW the oral presentation.	‐ Size and amount of TEXT READABLE AND USEFUL MOST OF THE TIME. SOMETIMES IT IS DIFFICULT TO READ. ‐ MOST IMAGES (NOT ALL) are WELL CHOSEN, and their QUALITY and SIZE MAKE THEM VISIBLE. ‐ Order of appearance of MOST of the elements CLARIFIES and HELPS to FOLLOW the oral presentation.	‐ The TEXT DOES NOT COMPLEMENT or DISTRACTS from the oral presentation. ‐ The IMAGES DO NOT COMPLEMENT the oral presentation. ‐ The order of appearance of the contents DOES NOT COMPLEMENT the oral presentation.	‐ ILLEGIBLE TEXT or EXCESSIVE QUANTITY. IT PREVENTS MAINTAINING ATTENTION. ‐ NON‐EXISTENT or POOR‐QUALITY images. ‐ The order of appearance of the elements DISTRACTS and PREVENTS FOLLOWING the oral presentation.
	**DOMAIN 6: INTERACTION OF THE PRESENTER WITH THE SUPPORTING MATERIAL (PRESENTATION)**			
	The presenter EXPLAINS and TIMELY POINTS OUT the elements of the presentation.	The presenter EXPLAINS and POINTS out the elements of the presentation appropriately MOST OF THE TIME.	The presenter LOOKS AT THE SCREEN EXCESSIVELY and RARELY EXPLAINS AND POINTS OUT the elements.	The presenter USES THE SCREEN AS A SCRIPT and DOES NOT EXPLAIN or POINTS OUT the elements.
	**DOMAIN 7: VERBAL COMMUNICATION (VOLUME AND TONE OF VOICE)**			
	‐ The presenter can be HEARD and FOLLOWED ALL THE TIME. ‐ The presenter ALWAYS EMPHASIZES the important aspects. ‐ The presenter DOES NOT USE FILLERS.	‐ The presenter can be HEARD and FOLLOWED MOST OF THE TIME (not always). ‐ The presenter EMPHASIZES important aspects MOST OF THE TIME (not always). ‐ The presenter SOMETIMES uses FILLERS.	‐ AUDIBLE VOLUME, however, the speech is MONOTONE. ‐ The presenter RARELY EMPHASIZES. ‐ The presenter USES FILLERS that OFTEN DISTRACT the audience.	‐ The presenter speaks TOO SLOWLY or FAST and CANNOT BE UNDERSTOOD. ‐ The presenter CONTINUOUSLY uses FILLERS that DISTRACT the audience.
	**DOMAIN 8: NON‐VERBAL COMMUNICATION**			
	‐ RELAXED and CONFIDENT posture. ‐ The body language HELPS to follow the presentation.	‐ The posture shows SOME NERVOUSNESS at times. ‐ OCCASIONAL DISTRACTING body language.	‐ The posture shows NERVOUSNESS MOST OF THE TIME. ‐ The body language MAKES it DIFFICULT to follow the presentation.	‐ The posture shows INSECURITY. ‐ The body language PREVENTS following the presentation.
	**DOMAIN 9: INTERACTION OF THE PRESENTER WITH THE AUDIENCE**			
	‐ The presenter INTERACTS and CONNECTS with the audience throughout the ENTIRE PRESENTATION. ‐ The presenter shows WILLINGNESS to COMMUNICATE throughout THE ENTIRE PRESENTATION.	‐ The presenter INTERACTS and CONNECTS with the audience MOST OF THE TIME (not always). ‐ The presenter shows WILLINGNESS to COMMUNICATE MOST OF THE TIME (not always).	‐ The presenter RARELY INTERACTS and CONNECTS with the audience. ‐ The presenter RARELY shows WILLINGNESS TO COMMUNICATE.	‐ The presenter DOES NOT INTERACT or CONNECT with the audience. ‐ The presenter DOES NOT show WILLINGNESS to COMMUNICATE.

### 2.3 Performance of the presentations

Six weeks later all groups performed before their peers and seven EDU. After each 10 min‐presentation, an extra period of 5 min was available for the presenters to answer questions asked by students from the rest of the groups or by EDU.

After each performance, all the EDU and the rest of the groups of students were asked to assess the presentations using the rubric. Students and EDU were assigned a randomized identifier number, blinded to the researchers and students, to guarantee the anonymity of the procedure. For this purpose, a faculty member who was not involved in the evaluation generated a list of randomized numbers and associated it with the complete lists of students and evaluators. Afterward, each student and EDU were individually informed of their identifying number. An online form with the nine evaluable items and the four levels of performance of the rubric were used for the assessment process. When filling out the forms, both students and EDU used their identifying numbers. Also, each student was asked to state whether it was their first time using the tool (non‐experienced students—NES) or whether they had used rubrics in their pre‐university years (experienced students—ES).

Numeric values of 4, 3, 2, and 1 were assigned to the levels of performance “excellent”, “satisfactory”, “needs improvement” and “needs much improvement” respectively for each evaluable item in the rubric. A final score was obtained for each assessment performed by either EDU or students of every presentation by calculating the average assigned score. All students were previously informed that the obtained results were to contribute partially to their team marks.

### 2.4 Satisfaction survey

After the presentation sessions were finished, students were asked to complete a satisfaction survey through a second online form using their identifier numbers. The questionnaire was designed following the guidelines for the development of questionnaires for educational research.^23^ In short, after reviewing the literature for a clear definition of the content and features to include some informal interviews with students from a previous cohort were conducted to establish adequate language to present the content. After this, a set of potential items was composed, trying to use clear, unambiguous, and understandable vocabulary for the students to ensure that the respondents would understand the content. This first draft was presented to colleagues and other faculty members with experience in conducting educational research studies to assess the representativeness, clarity, and relevance of the items. With the feedback obtained from the experts, certain items were corrected, and others were excluded. After this refinement, the document was presented to a small group of students, from whom more feedback was gathered to obtain the final version. The resulting document consisted of seven questions, four of them related to the rubric as an assessment tool and three to the rubric as a learning tool. The students were asked to state their opinions for both purposes (learning and assessment tool) regarding the usefulness of the rubric, the perceived sense of confidence provided by it, and the thoroughness of its contents. In addition, the student's perceptions regarding the easiness of use of the rubric as an assessment tool were also registered. Table 2 shows the questions included in the questionnaire. Students indicated their agreement according to a 5‐level Likert scale. EDU also completed the survey with their opinion regarding the rubric as an assessment tool.

**TABLE 2:** Data (in percentages of each group) obtained from the students’ and educators’ satisfaction survey.

Rubric as ASSESSMENT tool for students and educators.															
	Agree			Partially agree			Neither agree, nor disagree			Partially disagree			Disagree		
*The rubric…*	ES	NES	EDU	ES	NES	EDU	ES	NES	EDU	ES	NES	EDU	ES	NES	EDU
*…is easy to use*	90.2%	76.9%	100%	9.8%	19.2%	0%	0%	3.8%	0%	0%	0%	0%	0%	0%	0%
*…contains all the key aspects to evaluate the presentations*	78%	88.5%	85.7%	17.1%	11.5%	14.3%	0%	0%	0%	2.4%	0%	0%	2.4%	0%	0%
*…is useful to evaluate the presentation*.	70.7%	73.1%	100%	22%	15.4%	0%	4.9%	11.5%	0%	2.4%	0%	0%	0%	0%	0%
*… makes me feel more comfortable and confident to assess my peers*.	63.4%	61.5%	100%	26.8%	30.8%	0%	9.8%	7.7%	0%	0%	0%	0%	0%	0%	0%

Rubric as a LEARNING tool for students.															
*The rubric…*	ES	NES		ES	NES		ES	NES		ES	NES		ES	NES	
*…contains all the key aspects to prepare the presentation*.	68.3%	69.2%		29.3%	23.1%		0%	3.8%		2.4%	3.8%		0%	0%	
*…is useful to prepare the presentation*.	65.9%	50%		14.6%	26.9%		14.6%	15.4%		2.4%	0%		2.4%	7.7%	
*…makes me feel more comfortable and confident to perform the presentation*.	63.4%	46.2%		19.5%	36.4%		12.2%	11.5%		0%	3.8%		4.9%	3.8%	

### 2.5 Data and statistical analysis

The dependent variable related to the assigned scores from each group of students (ES or NES) and those from the EDU were compared with the Kruskall‐Wallis test as they violated the assumption of normal distribution.

The students’ perceptions regarding the rubric (ES or NES) were analyzed through the responses in the satisfaction survey with the Chi‐squared test.

## 3 RESULTS

Sixty‐seven out of the 86 undergraduate students completed the whole process (assessment and satisfaction survey). 41 scholars stated having had a previous experience with the use of rubrics in the pre‐university courses (ES) whereas 26 had not (NES).

### 3.1 Scores assigned

No differences were found between the total scores assigned to their peers by ES (mean = 3.65, standard deviation [SD] = 0.38) and NES (mean = 3.66, SD = 0.40). Both graded the presentations significantly higher than EDU (mean = 3.25, SD = 0.50).

Regarding the scores assigned to each presentation, no differences among ES, NES, and EDU were found in seven out of sixteen presentations. ES and NES graded higher than EDU in seven out of sixteen presentations. In one presentation NES graded significantly higher than EDU, and in another presentation, ES graded significantly higher than EDU. Although EDU assigned lower scores than students in all nine domains of the rubric, there were three of them where these differences were more evident: “interaction with the supporting materials”, “organization” and “interaction with the audience”.

### 3.2 Satisfaction survey: rubric as an assessment tool

Data obtained from the students’ satisfaction survey are shown in Table 2. Most students considered the rubric an easy and useful assessment tool and found that it contained all the key aspects to comprehensively evaluate the presentation. 63.4% of ES and 61.5% of NES considered that the rubric provided them with confidence when assessing their peers. Another 26.8% of ES and 30% of NES also stated that they had benefited from these properties at least partially. No statistically significant differences were found between ES and NES. With no statistical differences between students and EDU, the latter also considered the rubric easy to use, useful, and a tool that provides confidence to the evaluator (100%). 85.7% of EDU considered the rubric to contain all the key aspects to evaluate oral presentations.

### 3.3 Satisfaction survey: rubric as a learning tool

When addressing students’ opinions about the rubric as a learning tool, although most of them found the rubric useful and contained all the key aspects, the percentages of ES and NES that totally agreed with these affirmations were lower than when considering it solely as an assessment tool. The rubric improved the level of confidence of 63.4% of ES and 46.2% of NES when preparing their presentations, with no statistically significant differences between ES and NES.

## 4 DISCUSSION

Oral presentations have been proposed as training activities in dental curricula to acquire communicative skills.^3^ Due to its simplicity, this methodology can be suitable for a first approach in the practice of communication, as students prepare a pre‐determined topic and expose it in front of an audience (peers and educators in our study) in a limited amount of time. They contrast with other advanced activities also included in dental school programs such as the use of debates on certain topics^24^, ^25^ that may require a wider knowledge and the use of other crucial soft skills developed during the degree as critical thinking^26^ and might be more suitable for superior years. For the assessment of these activities, rubrics seem to be an adequate tool as they explicitly present pre‐established objectives and standardized criteria.

The present study analyzed the effect of previous experience in the use of rubrics on the perceptions and scores assigned by students in peer assessment of oral presentations. No differences were found among ES and NES and therefore the null hypothesis could not be rejected. The authors associate this finding with the previous contact of students with the new rubric, as they all were introduced to it, and they were able to handle it during the preparation of the presentations. Furthermore, to obtain more realistic results, they were informed that their obtained scores would be taken into consideration as part of the evaluation of the activity. The introduction and explanation during the initial session and having access to it while working on their tasks may have contributed to minimizing differences in the understanding of this tool for peer assessment and ES and NES performed similarly. The benefits of previous contact with rubrics before using have been described earlier.^27^

Comparation with EDU was introduced as a reference, for a clearer sign of overall understanding of the assessment task. EDU assigned significantly lower scores than students when evaluating their peers, which led to the rejection of the second null hypothesis. This result has also been described by other authors.^28^ Discrepancies between scores of students and educators might be attributed to different backgrounds. In the present study, major differences between students and EDU were found in the domain of “organization”, “interaction with the supporting materials” and “interaction with the audience”. This finding could be related to the different levels of expertise of both groups when trying to structure an oral presentation to convey a message to the audience, optimizing the use of the application to compose presentations, or creating a dialogue with the attendants to maintain the attention during the whole communicative process. Familiarity with these strategies by EDU could make them more critical when evaluating these aspects. These differences could be also related to the methods and standards followed by educators to design the rubric^29^, ^30^, ^31^ and the interpretation by the students of the descriptions of performance comprised in it. Sharing the construction of assessment tools between students and educators has been proposed as a strategy that could palliate these understanding issues and increase their level of engagement.^32^ Another described advantage of this collaborative production of evaluation tools is that it might improve the communication skills of the student with peers and educators.^33^ Nevertheless, some authors recommend avoiding this method with first‐year students,^32^ as their experience with higher studies is limited and their expectations related to academic competencies could be unrealistic.^34^ Moreover, this process requires a high amount of time which limits its applications in all situations or periods during the academic course.^32^ The reason for selecting first‐year students for this study is the fact that the use of rubrics is more common in higher education programs, and many university students have contact with them in their initial years. Thus, students needed to be at the beginning of the program, to increase the chance of including a sample of students lacking previous experience with rubrics. Other authors also consider a previous practice with mock assignments necessary if the evaluators had not had previous experience.^21^ According to the results of this study, it seems that this previous practice may have not been necessary for minimizing the effect of lack of experience with rubrics in general, being substituted by familiarization during the preparation process of the oral presentation. However, prior calibration of the students and guidance in the interpretation of the specific assessment criteria with an initial example may have helped reduce the discrepancies between scores assigned by EDU and students. Additional examples of what should be considered relevant, logically ordered, or well‐chosen could reduce subjectivity and lead to a deeper understanding of the expected performance. Further studies including initial training with sample tasks need to be conducted to validate this assumption. Also, comparisons with EDU familiar and unfamiliar with this specific rubric may have helped evaluate the overall clarity and understandability of the criteria. Another factor that may influence the difference between EDU and students is the fact that students assessing their peers may be biased by personal relationships among them, which could lead to higher assigned scores.^35^ Strategies such as anonymous evaluation systems^36^ have been proposed to reduce the impact of friendship in the assigned scores. In this study it was not possible to implement a double‐blinded evaluation system due to the essence of the activity, however, the authors consider that subjectivity could be minimized because of the evaluation tool provided. Also, the fact that the students were randomly assigned to the different work groups could have helped reduce the effect of the relationships among them.

First‐year students might be immersed in the process of discovering university life and creating bonds with their classmates, a fact that might have affected the scores assigned to their peers. Nevertheless, this weak point was expected to be present in both ES and NES groups. The aim of this study was not to analyze this situation but the use of an assessment tool by students who had had previous experience (in pre‐university courses) and by those who had not, in order to enlighten the process in which the students learn to use an evaluation resource. For this reason, first‐year students were selected as the authors were aware that not all of them had used evaluation rubrics previously.

Most of the participants in this study found the rubric beneficial during the assessment process as they considered it easy to use, and useful and found that it contained all the key aspects to evaluate the presentations. Other authors have described similar benefits when students use evaluating rubrics during their university years.^15^, ^37^ In this study about 90% of the students felt that this tool made them feel more confident (totally or partially) both during the assessment task and the performance of the activity (more than 80%). This fact could be relevant to improve the experience of the students in academically demanding tasks such as delivering oral presentations in their first university year and to increase the level of engagement. In contrast, other studies have registered fewer positive results in students’ feedback and reported that some students considered them restrictive or lacking clarity.^38^, ^39^ Nevertheless, in this study, no differences were found between the perceptions expressed by ES and NES. The authors of this study believe that this fact might be explained by the structure and characteristics of the new rubric and by the previous familiarization with the tool during the preparation period, which could have increased the level of knowledge and understanding of it and consequently palliated the differences between ES and NES. According to students’ opinions, using the rubric helped enhance their confidence during the evaluation process. Students seemed to be satisfied with the usefulness of the rubric in preparing the oral presentations. It has been previously reported that the incorporation of rubrics might be beneficial for the overall performance of students in a specific task.^40^ A limitation of the present study is that no comparison was made with a group of students who did not use the rubric, and therefore the sense of improved confidence and performance is based on the subjective perceptions of students after using the assessment tool, and not on objective measurement and comparation with a control group. For ethical reasons, the design of the study did not contemplate excluding students from the previous introduction of the assessment criteria, and therefore the only possible data to assess the overall usefulness of the tool, regarding performance and confidence was to assess the students’ own perceptions.

Perceptions of the rubric as a learning tool were not as positive as looking at it as an assessment tool. This finding could be explained by the fact that the rubric carefully describes the criteria that define the different levels of performance, but it fails to provide the students with precise guidelines to achieve a satisfactory execution. Thus, though conceived as a formative instrument, it might be somewhat limited in providing specific indications for improving the performance of the students. An additional strategy to help students acquire the desired skills could be providing supplementary resources such as written or multimedia material. After considering the results of the present study, the authors have started to work on elaborating additional materials to complement the rubric in communication training. Subsequent comparative studies to evaluate the different approaches and methods may be the best tactic to determine the most effective procedure to teach communicative skills.

## 5 CONCLUSIONS

Considering the limitations of the present study, it can be concluded that the previous experience of students in the use of rubrics does not influence the scores that they assign to their peers on oral presentations, however, both groups tend to grade their peers with higher scores than educators. The use of the newly proposed rubric helps the students feel confident and comfortable, at least partially, during the preparation of the activity and the assessment process.

## CONFLICT OF INTEREST STATEMENT

The authors declare no conflict of interest.
